# Use of Electronic Patient Messaging by Pregnant Patients Receiving Prenatal Care at an Academic Health System: Retrospective Cohort Study

**DOI:** 10.2196/51637

**Published:** 2024-04-29

**Authors:** Kai Holder, Joe Feinglass, Charlotte Niznik, Lynn M Yee

**Affiliations:** 1 Feinberg School of Medicine, Northwestern University, Chicago, IL, United States; 2 Division of General Internal Medicine and Geriatrics, Feinberg School of Medicine, Northwestern University, Chicago, IL, United States; 3 Division of Maternal–Fetal Medicine, Department of Obstetrics and Gynecology, Northwestern Medicine, Chicago, IL, United States

**Keywords:** patient portal, secure messaging, telehealth, health literacy, health disparities, disparity, disparities, telemedicine, information and communication technology, ICT, portals, messaging, use, technology use, pregnant, pregnancy, maternal, obstetric, obstetrics, prenatal, antenatal, demographic, demographics

## Abstract

**Background:**

The COVID-19 pandemic accelerated telemedicine and mobile app use, potentially changing our historic model of maternity care. MyChart is a widely adopted mobile app used in health care settings specifically for its role in facilitating communication between health care providers and patients with its messaging function in a secure patient portal. However, previous studies analyzing portal use in obstetric populations have demonstrated significant sociodemographic disparities in portal enrollment and messaging, specifically showing that patients who have a low income and are non-Hispanic Black, Hispanic, and uninsured are less likely to use patient portals.

**Objective:**

The study aimed to estimate changes in patient portal use and intensity in prenatal care before and during the pandemic period and to identify sociodemographic and clinical disparities that continued during the pandemic.

**Methods:**

This retrospective cohort study used electronic medical record (EMR) and administrative data from our health system’s Enterprise Data Warehouse. Records were obtained for the first pregnancy episode of all patients who received antenatal care at 8 academically affiliated practices and delivered at a large urban academic medical center from January 1, 2018, to July 22, 2021, in Chicago, Illinois. All patients were aged 18 years or older and attended ≥3 clinical encounters during pregnancy at the practices that used the EMR portal. Patients were categorized by the number of secure messages sent during pregnancy as nonusers or as infrequent (≤5 messages), moderate (6-14 messages), or frequent (≥15 messages) users. Monthly portal use and intensity rates were computed over 43 months from 2018 to 2021 before, during, and after the COVID-19 pandemic shutdown. A logistic regression model was estimated to identify patient sociodemographic and clinical subgroups with the highest portal nonuse.

**Results:**

Among 12,380 patients, 2681 (21.7%) never used the portal, and 2680 (21.6%), 3754 (30.3%), and 3265 (26.4%) were infrequent, moderate, and frequent users, respectively. Portal use and intensity increased significantly over the study period, particularly after the pandemic. The number of nonusing patients decreased between 2018 and 2021, from 996 of 3522 (28.3%) in 2018 to only 227 of 1743 (13%) in the first 7 months of 2021. Conversely, the number of patients with 15 or more messages doubled, from 642 of 3522 (18.2%) in 2018 to 654 of 1743 (37.5%) in 2021. The youngest patients, non-Hispanic Black and Hispanic patients, and, particularly, non–English-speaking patients had significantly higher odds of continued nonuse. Patients with preexisting comorbidities, hypertensive disorders of pregnancy, diabetes, and a history of mental health conditions were all significantly associated with higher portal use and intensity.

**Conclusions:**

Reducing disparities in messaging use will require outreach and assistance to low-use patient groups, including education addressing health literacy and encouraging appropriate and effective use of messaging.

## Introduction

As health systems have adopted electronic health records, patient portal platforms, including widely used mobile apps such as MyChart, have proliferated in both primary care and medical specialties, including obstetrics [1,2]. A patient portal is a secure web-based interface connected to the personal electronic health record. Through the patient portal, individuals are able to review their health record, schedule appointments, refill prescriptions, and conduct secure direct messaging with health care providers [3]. The advent of mobile apps such as MyChart, recognized as the number-one medical app in terms of downloads on the Apple App Store and amassing more than 10 million downloads on Google Play, underscores the portability of these features, facilitating secure communication between patients and health care providers. Patient portals, augmented by mobile apps such as MyChart, provide patients with a resource where they can readily communicate with providers and actively participate in their health care [4]. The COVID-19 pandemic has accelerated the use of telemedicine and eHealth, potentially changing our historic model of maternity care [5].

Patient portal use has been steadily increasing since the 2009 Health Information Technology for Economic and Clinical Health (HITECH) Act and government financial incentives motivating adoption of required technology such as electronic patient portals [6]. Since patient portals have become more common in medical care, several studies have analyzed the effects of patient portals on clinical outcomes. In outpatient, primary care practices, the nonobstetrics literature has provided evidence that electronic portal use is associated with improved patient satisfaction and patient-provider communication [6-9]. Secure messaging within electronic patient portals has also been associated with positive clinical outcomes; for instance, diabetes management patients who used a secure messaging feature were found to have lower hemoglobin A_1c_ values [6,8-15]. Additionally, pilot studies analyzing portal use in obstetric populations have suggested that portals can be a useful tool for management of complex medical comorbidities. A study evaluating portal use and glucose control in an obstetric population found that patients who were active portal users were less likely to have within-goal glycemic control, suggesting that pregnant patients with suboptimal glycemic control may have been more readily engaged in secure messaging with providers [16]. However, previous studies analyzing portal use in obstetric populations have demonstrated significant sociodemographic disparities in portal enrollment and messaging, specifically showing that patients who are non-Hispanic Black, Hispanic, uninsured, and have low income were less likely to enroll in and use patient portals [15]. The rapid expansion of patient portals raises concern that disparities in health outcomes and health care access may be further exacerbated by disparities in technology access and electronic health literacy, which has been demonstrated in prior research [5,6]. Because of findings from previous studies that have suggested associations between patient portal use and improved clinical outcomes, there is a need to gain a deeper understanding of use patterns and the factors influencing the use of patient portals during pregnancy [6-16].

It was therefore of interest to evaluate our health system’s use of an obstetric patient portal in the context of the rapid expansion of telemedicine since the start of the COVID-19 pandemic in 2020. This study aimed to estimate changes in patient portal use and intensity in prenatal care before and during the pandemic period and to identify sociodemographic and clinical disparities that continued during the pandemic.

## Methods

### Overview

This was a retrospective cohort study using electronic medical record (EMR) and administrative data from our health system‘s Enterprise Data Warehouse. Records were obtained for the first pregnancy episode of all patients who received antenatal care at 8 academically affiliated practices and delivered at a large urban academic medical center from January 1, 2018, to July 22, 2021, in Chicago, Illinois. The study included all patients aged 18 years or older who attended at least 3 clinical encounters during pregnancy at the practices that used the EMR portal.

### Ethical Considerations

The study was approved by the Institutional Review Board of Northwestern University with waiver of informed consent (STU00202847). This study follows the STROBE (Strengthening the Reporting of Observational Studies in Epidemiology) guidelines for reporting observational research.

### Trends in Patient Portal Use and Message Frequency

Our health system faculty practices use the EpicCare EMR and MyChart, the associated commercial patient portal. Patients can view records, review laboratory and imaging results, send messages to providers, schedule appointments, and request medication refills via MyChart. During a clinical encounter, a provider can generate an individualized access code designated for portal enrollment, or patients can self-enroll in the portal via email without a prior access code. To gain access to portal use and functions, patients must activate their MyChart account through the portal website. The patient can then access MyChart through web-based interfaces or mobile apps. A formal mechanism for declining the MyChart invitation is unavailable, and all patients are considered to be enrolled in the portal; thus, we were unable to analyze patients who were provided with a MyChart invitation but chose not to use the portal.

Only patient portal use for communication with obstetric providers (physicians, nurse practitioners, certified nurse midwives, or nurses within the Department of Obstetrics and Gynecology) was considered active use for this study. Communication with nonprenatal care providers was excluded from this analysis, as we solely analyzed messaging use within the patients’ prenatal practice. Patients were considered enrolled in the portal if they had an account at the time of delivery. Portal enrollment was not specifically analyzed in this study. Patients were considered portal users if they sent at least 1 secure message during pregnancy. Portal users were further categorized by intensity, which was classified by the number of secure messages sent during pregnancy as infrequent (≤5 messages), moderate (6-14 messages), or frequent (≥15 messages). The categorization of portal use into infrequent, moderate, or frequent categories was determined by assessing patient portal use patterns within our clinic’s prenatal practices. Patients sending fewer than 5 secure messages were classified as infrequent users, signifying limited engagement with the portal. Those sending between 6 and 14 messages were categorized as moderate users, indicating a moderate level of portal interaction. Patients sending more than 15 secure messages were considered frequent users, reflecting active and regular portal engagement. These specific cutoff points were chosen to distinguish between different levels of patient portal engagement. Our data did not permit identification of “threads” across messages involving multiple messages on the same issue, as this would require natural language processing of message texts that could identify specific issue content and the duration of threads across a given prenatal time interval.

### Patient Portal Use by Patient Sociodemographic and Clinical Characteristics

Patient sociodemographic and clinical data were categorized using hospital administrative data and *International Classification of Diseases, Tenth Revision* (*ICD-10*) diagnosis and procedure codes, as well as prenatal visits, characterized as <9, 9-12 or >12 visits. Maternal age was categorized as <20, 20-24, 25-29, 30-34, 35-39, and ≥40 years at the time of prenatal care enrollment. Race and ethnicity were categorized as Hispanic, non-Hispanic Black, non-Hispanic White, Asian, and other/unknown. Additional sociodemographic characteristics that were collected included preference for a non-English language, antenatal care insurance status (Medicaid versus private or other), and residential zip code. Maternal residential zip codes in Illinois were matched to census zip code tabulation areas (ZCTAs) using the 2020 American Community Survey for the percentage of households living at or below the poverty level. Individuals were categorized as living in ZCTAs with <5%, 5%-9.99%, 10%-19.99%, or >20% of households living at or below the poverty level, or being non–Illinois residents [17].

In addition to multifetal gestation and prior cesarean delivery, parity was categorized as either nulliparous, 1 pregnancy, or 2 or more pregnancies. Maternal BMI at birth was categorized as normal weight (≤24 kg/m^2^), overweight (25-29 kg/m^2^), or obese (≥30 kg/m^2^). We used *ICD-10* data from the delivery admission to characterize the prevalence of any of a number of chronic conditions, including cardiac disease, bleeding disorder, pulmonary hypertension, chronic renal disease, gastrointestinal disease, HIV/AIDS, bariatric surgery, asthma, connective tissue or autoimmune disease, neuromuscular disease, and thyrotoxicosis. We also categorized patients as having a history of depression or severe mental illness (schizophrenia, bipolar disorder, or psychosis) or substance use. We found that hospital *ICD-10* coders had incorrectly coded *both* gestational and preexisting diabetes and hypertension for a substantial number of delivery admissions. We therefore characterized diabetes and hypertension codes as either uniquely preexisting only, gestational only, or coded for both.

### Statistical Analysis

Monthly messaging intensity rates were computed to assess changes before and after the March 2020 pandemic. We determined the significance of bivariate differences in messaging frequency using *χ*^2^ tests. We estimated a logistic regression model of the likelihood of zero portal use for patients who delivered from April 2020 during the 15 months after the end of the pandemic in March 2022. Analyses were performed using SPSS Statistics (version 28; IBM Corp).

## Results

### Trends in Portal Use and Message Frequency

**Figure 1.**
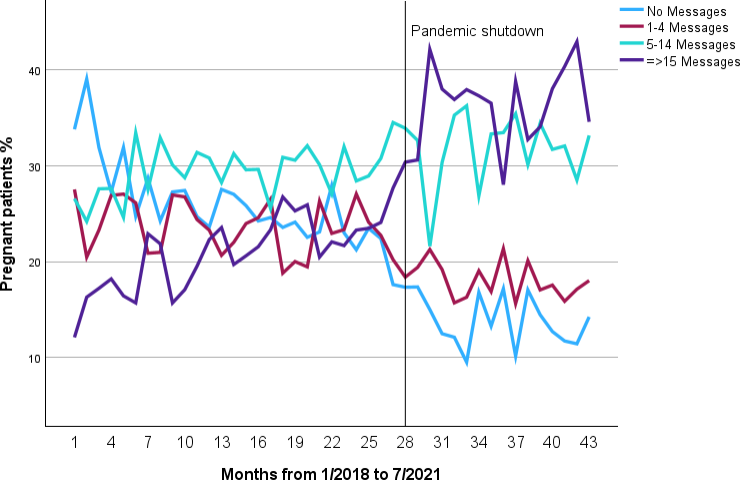
Pregnant patient portal messaging intensity. There were 12,380 patients with at least 3 prenatal visits with deliveries between January 1, 2018, and July 22, 2021.

A total of 12,380 patients were eligible for inclusion. In the total study period, 2681 (21.7%) of patients were nonusers of the portal, 2680 (21.6%) were infrequent users, 3754 (30.3%) were moderate users, and 3265 (26.4%) were frequent users. Figure 1 displays portal use rates by delivery date over the 43-month study period, denoting April 2020 (month 28) as the full onset of the COVID-19 pandemic. There was a modest trend toward increased use in the first study years, followed by a rapid increase in use and a correspondingly dramatic reduction in nonuse after the onset of the COVID-19 pandemic. The number of patients who were nonusers decreased between 2018 and 2021, from 996 of 3522 (28.3%) in 2018 to only 227 of 1743 (13%) in the first 7 months of 2021. Conversely, the number of patients with 15 or more messages doubled, from 642 of 3522 (18.2%) in 2018 to 654 of 1743 ( 37.5%) in 2021.

### Patient Portal Use by Sociodemographic and Clinical Characteristics

Table 1 displays patient characteristics by messaging intensity. The proportion of those with no portal use was correlated with the number of prenatal visits. Of the 2430 of 12,380 (19.6%) study patients with 8 or fewer visits, 1123 (46.2%) were nonusers overall, as compared to only 488 (10.8%) nonusers among 4520 patients (36.5%) in the sample with 13 or more visits. Those with the most prenatal visits had over 3 times the proportion of frequent messaging than those with the fewest visits. Higher parity was similarly correlated with lower message intensity.

**Table 1.** Obstetric patient electronic medical record portal use by number of messages sent during pregnancy among patients with at least 3 prenatal visits. There were 12,380 patients with deliveries from January 1, 2018, to July 22, 2021. All comparison were at *P*<.001 except preeclampsia (*P*=.03).

			Total, n (%)	No messages, n (%)^a^	≤5 messages, n (%)^a^	6-14 messages, n (%)^a^	≥15 messages, n (%)^a^
Overall			12,380 (100)	2681 (21.7)	2680 (21.6)	3754 (30.3)	3265 (26.4)
**Delivery year**							
	2019-2020		3522 (28.4)	996 (28.3)	863 (24.5)	1021 (29)	642 (18.2)
	2020-2021		4274 (34.5)	1038 (24.3)	984 (23)	1267 (29.6)	985 (23)
	2021-7/2022		4584 (37)	647 (14.1)	833 (18.2)	1466 (32)	1638 (35.7)
**Prenatal visits (n)**							
	<9		2430 (19.6)	1123 (46.2)	523 (21.5)	514 (21.2)	270 (11.1)
	9-12		5430 (43.9)	1070 (19.7)	1289 (23.7)	1709 (31.5)	1362 (25.1)
	>12		4520 (36.5)	488 (10.8)	868 (19.2)	1531 (33.9)	1633 (36.1)
**Route of delivery**							
	Vaginal delivery		9235 (74.6)	1974 (21.4)	2071 (22.4)	2865 (31)	2325 (25.2)
	Cesarean section		3145 (25.4)	707 (22.5)	609 (19.4)	889 (28.3)	940 (29.9)
Prior cesarean section			1781 (14.4)	509 (28.6)	379 (21.3)	494 (27.7)	399 (22.4)
Multiple gestation			448 (3.6)	118 (26.3)	92 (20.5)	116 (25.9)	122 (27.2)
**Parity (n)**							
	0		7266 (58.7)	1088 (15)	1451 (20)	2366 (32.6)	2361 (32.5)
	1		3458 (27.9)	891 (25.8)	849 (24.6)	1026 (29.7)	692 (20)
	≥2		1656 (13.4)	702 (42.4)	380 (22.9)	362 (21.9)	212 (12.8)
**Sociodemographic characteristics**							
	**Age (years)**						
		<20	74 (0.6)	52 (70.3)	18 (24.3)	2 (2.7)	2 (2.7)
		20-24	601 (4.9)	325 (54.1)	132 (22)	80 (13.3)	64 (10.6)
		25-29	1876 (15.2)	584 (31.1)	454 (24.2)	499 (26.6)	339 (18.1)
		30-34	5263 (42.5)	934 (17.7)	1155 (21.9)	1753 (33.3)	1421 (27)
		35-39	3693 (29.8)	624 (16.9)	758 (20.5)	1185 (32.1)	1126 (30.5)
		≥40	873 (7.1)	162 (18.6)	163 (18.7)	235 (26.9)	313 (35.9)
	**Race/ethnicity**						
		Asian/Pacific Islander	1264 (10.2)	212 (16.8)	243 (19.2)	429 (33.9)	380 (30.1)
		Hispanic	1616 (13.1)	572 (35.4)	365 (22.6)	388 (24)	291 (18)
		Non-Hispanic Black	1770 (14.3)	778 (44)	365 (20.6)	355 (20.1)	272 (15.4)
		Non-Hispanic White	6238 (50.4)	832 (13.3)	1355 (21.7)	2111 (33.8)	1940 (31.1)
		Other/unknown	1492 (12.1)	287 (19.2)	352 (23.6)	471 (31.6)	382 (25.6)
	Medicaid		1730 (14)	1095 (63.3)	349 (20.2)	179 (10.3)	107 (6.2)
	Non–English speaking		400 (3.2)	(44.8)	(18)	(23)	(14.2)
	**Zip-code–level household poverty**						
		<5%	4853 (39.2)	721 (14.9)	1000 (20.6)	1679 (34.6)	1453 (29.9)
		5%-9.99%	2973 (24)	568 (19.1)	667 (22.4)	927 (31.2)	811 (27.3)
		10%-19.99%	3105 (25.1)	863 (27.8)	710 (22.9)	809 (26.1)	723 (23.3)
		≥20%	1077 (8.7)	460 (42.7)	229 (21.3)	214 (19.9)	174 (16.2)
		Non-Illinois resident	372 (3)	69 (18.5)	74 (19.9)	125 (33.6)	104 (28)
**Clinical characteristics**							
	**BMI at delivery (kg/m^2^)**						
		<18.5	1377 (11.1)	260 (18.9)	292 (21.2)	430 (31.2)	395 (28.7)
		18.5-29.9	4793 (38.7)	848 (17.7)	1105 (23.1)	1542 (32.2)	1298 (27.1)
		>30	5498 (44.4)	1383 (25.2)	1136 (20.7)	1584 (28.8)	1395 (25.4)
	Anemia		1559 (12.6)	451 (28.9)	302 (19.4)	392 (25.1)	414 (26.6)
	**Diabetes**						
		Preexisting diabetes	291 (2.4)	70 (24.1)	41 (14.1)	71 (24.4)	109 (37.5)
		Gestational diabetes	1087 (8.8)	128 (11.8)	155 (14.3)	354 (32.6)	450 (41.4)
		Preexisting diabetes and gestational diabetes	332 (2.7)	63 (19)	36 (10.8)	96 (28.9)	137 (41.3)
	**Hypertension**						
		Preexisting hypertension	672 (5.4)	185 (27.5)	126 (18.8)	155 (23.1)	206 (30.7)
		Gestational hypertension	677 (5.5)	168 (24.8)	128 (18.9)	194 (28.7)	187 (27.6)
		Preexisting hypertension and gestational hypertension	316 (2.6)	83 (26.3)	46 (14.6)	89 (28.2)	98 (31)
	Preeclampsia		141 (1.1)	39 (27.7)	25 (17.7)	31 (22)	46 (32.6)
	Preexisting comorbidity^b^		1973 (15.9)	400 (20.3)	409 (20.7)	544 (27.6)	620 (31.4)
	History of depression or severe mental illness		3950 (31.9)	666 (16.9)	729 (18.5)	1193 (30.2)	1362 (34.5)
	Substance use		312 (2.5)	113 (36.2)	55 (17.6)	69 (22.1)	75 (24)

a Percentage calculated against the total for each characteristic.

b Preexisting comorbidities included cardiac disease, bleeding disorder, pulmonary hypertension, chronic renal disease, gastrointestinal disease, HIV/AIDS, bariatric surgery, asthma, connective tissue or autoimmune disease, neuromuscular disease, thyrotoxicosis, history of depression or severe mental illness (schizophrenia, bipolar disorder, or psychosis), or substance use.

Portal use was more frequent among those who were older, with the lowest messaging intensity among the youngest patients (younger than 25 years), who represented only 675 (5.5%) of all births. The highest messaging intensity was among patients older than 30 years. Non-Hispanic Black patients had the highest proportion of nonuse at 783 of 1784 (44%), followed by Hispanic patients at 581 of 1629 (35.4%) and non–English-speaking patients at 180 of 401 (44.8%). While patients with Medicaid comprised only 14% of the patient population, they had the highest proportion of nonuse among all patient subgroups at 1095 of 1730 (63.3%), and there was a clear poverty-level gradient in messaging intensity across ZCTAs.

**Figure 2.**
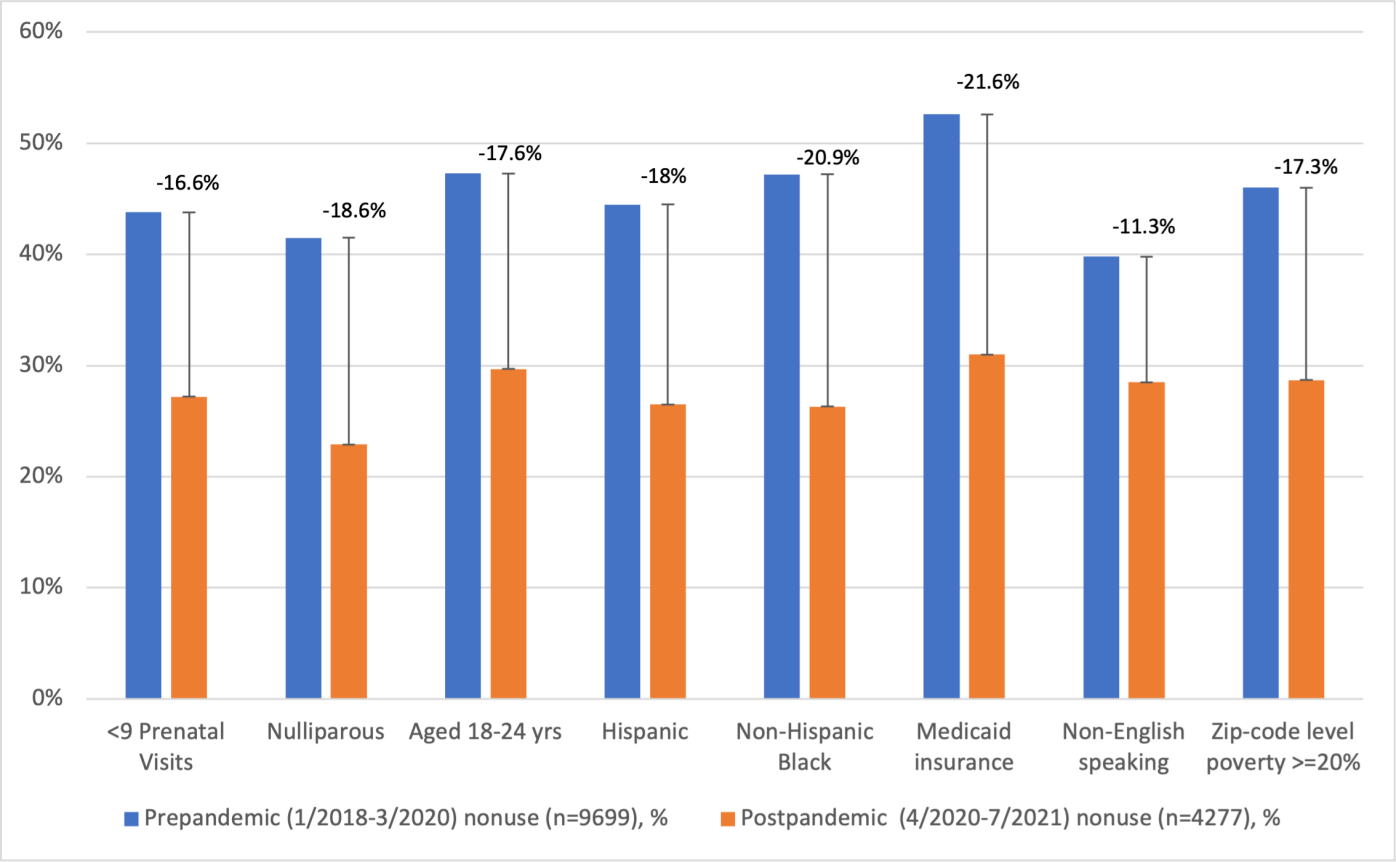
Change in the proportion of patients with highest portal nonuse after the April 2020 pandemic shutdown.

Figure 2 presents change in portal use over time for patients with sociodemographic and clinical characteristics associated with high rates of portal nonuse who delivered after April 2020. Patients with Medicaid births had a 21.6% reduction in their nonuse rate after the pandemic. Similar large increases in use occurred among all the groups profiled, including those with fewer prenatal visits, as well as younger, minority, and lower-income patients.

### Odds of Pandemic Portal Nonuse

Our logistic model of nonuse, based on the 4277 patients with postpandemic deliveries, showed that the number of visits and parity remained strongly correlated with the likelihood of portal nonuse (Table 2). Those with <8 visits were 97% more likely to have been nonusers than those with >12 prenatal visits, and patients with ≥2 parity were over 3 times more likely to be nonusers than nulliparous patients. Confirming bivariate results for the whole period, the youngest patients, non-Hispanic Black patients, and Hispanic patients, particularly non–English speaking patients, had the highest odds of continued nonuse. Patients with gestational diabetes were significantly more likely to be portal users, while patients with other comorbid conditions and those with preexisting mental health conditions were also more likely to use the portal.

**Table 2.** Logistic regression results for postpandemic obstetric patient nonuse of electronic medical record portal messaging among patients (n=4277) with deliveries from April, 2020, to July 22, 2021.

Characteristics			Odds ratio (95% CI)
**Sociodemographic characteristics**			
	**Age (years)**		
		<20	3.58 (2.02-6.33)
		20-24	1.90 (1.50-2.40)
		25-29	1.24 (1.06-1.45)
		30-34	Reference
		35-39	0.90 (0.79-1.03)
		≥40	0.84 (0.67-1.06)
	**Race/ethnicity**		
		Asian/Pacific Islander	1.00 (0.82-1.22)
		Hispanic	1.58 (1.33-1.87)
		Non-Hispanic Black	1.83 (1.52-2.20)
		Non-Hispanic White	Reference
		Other/unknown	1.18 (0.99-1.40)
	Medicaid insurance		4.27 (3.64-5.01)
	Non–English speaking		2.52 (1.93-3.31)
	**Zip-code–level household poverty**		
		<5%	Reference
		5%-9.99%	1.03 (0.89-1.19)
		10%-19.99%	1.14 (0.98-1.31)
		≥20%	1.15 (0.93-1.43)
		Non–Illinois resident	1.00 (0.73-1.37)
**Clinical characteristics**			
	Anemia		0.95 (0.81-1.12)
	**BMI at delivery (kg/m^2^)**		
		<18.5	Reference
		18.5-29.9	0.87 (0.74-1.0)
		≥30	0.96 (0.82-1.12)
	**Diabetes coding**		
		Preexisting diabetes	0.77 (0.53-1.12)
		Gestational diabetes	0.34 (0.27-0.43)
		Preexisting diabetes and gestational diabetes	0.63 (0.44-0.90)
	**Hypertension coding**		
		Preexisting hypertension	1.16 (0.91-1.79)
		Gestational hypertension	1.11 (0.88-1.41)
		Preexisting hypertension and gestational hypertension	1.09 (0.86-1.38)
		Preeclampsia	0.94 (0.58-1.54)
		Preexisting comorbidity^a^	0.81 (0.69-0.94)
		History of depression or severe mental illness	0.69 (0.61-0.78)

a Preexisting comorbidities included cardiac disease, bleeding disorder, pulmonary hypertension, chronic renal disease, gastrointestinal disease, HIV/AIDS, bariatric surgery, asthma, connective tissue or autoimmune disease, neuromuscular disease, thyrotoxicosis, history of depression or severe mental illness (schizophrenia, bipolar disorder, or psychosis), or substance use.

## Discussion

### Principal Findings

This study was undertaken to guide efforts to improve prenatal portal use by addressing what were already known to be significant socioeconomic disparities in use. We found that messaging frequency significantly increased over the 43-month study period. Most notably, there was a decline in nonuse in the immediate postpandemic period, approximately cutting in half the previous nonuser population from 25.8% to 13.9%. However, pandemic nonuse remained concentrated among patients with fewer prenatal visits, higher parity, and public insurance, as well as those who were younger, were not English speakers, had lower income, and identified as non-Hispanic Black or Hispanic. As hypothesized, patients with high-risk clinical conditions, such as history of depression or severe mental illness, preexisting comorbidities, or gestational or preexisting diabetes, were more likely to use the portal and to be frequent messengers.

The socioeconomic disparities found in this cohort are consistent with prior literature analyzing patient portal use and messaging in obstetric and nonobstetric populations. It has been suggested in several studies that those who identify as non-White, have low income, and are publicly insured are less likely to use portals than their counterparts [5,15,18-22]. Numerous studies have demonstrated that non-Hispanic Black, Hispanic, low-income, and publicly insured or uninsured patients were less likely to activate and use portal messaging. A study of more than 1700 primary care patients receiving care at Kaiser Permanente Georgia showed that compared to non-Hispanic White participants, non-Hispanic Black patients were less likely to register for electronic patient portals, even after controlling for differences in education, income, or internet access, which were also associated with portal registration [4,15]. Previous studies have shown conflicting conclusions regarding the associations of high-risk clinical characteristics and portal use intensity [10,23-25]. For example, 2 studies in a primary care setting found that patients with chronic conditions and conditions associated with increased morbidity used the portal more often than healthier patients [4,24]. However, another study in a similar primary care setting conversely found that patients with fewer medical problems used the portal more often than patients with chronic conditions [25].

### Potential Value of Increasing Patient Portal Use in Obstetrics

During the COVID-19 pandemic, health care providers increasingly relied on telehealth and electronic communication methods for health care delivery and patient care [5,19,20]. Telehealth and electronic patient communication were especially important for obstetrics, due to the necessity to continue scheduled prenatal care visits and screenings. The findings in this study demonstrate that messaging may have become a more important care function in the postpandemic period, despite the return to in-person visits.

The antenatal period represents a critical time, when health care engagement may influence both maternal and infant outcomes; however, disparities in antenatal portal use have been identified. Several studies that analyzed portal use in the setting of obstetrics demonstrate greater portal use in antenatal patients who have chronic conditions and are at higher risk for pregnancy complications; however, other studies suggest that medically high-risk obstetric patients were less likely to enroll and use portals [15,16,26]. This cohort’s findings align with the former, demonstrating that participants with high-risk clinical conditions, including preexisting comorbidities, preexisting mental illness, and gestational diabetes, were more likely to use the portal and use the portal frequently.

### Implications for Improving Patient Communication With Providers in Obstetrics

With secure messaging, patients can discuss a variety of topics, such as a change in condition, a new condition, laboratory results, and prescription concerns. A clinical example of the impact of secure messaging is the management of gestational diabetes mellitus. In this patient population, secure messaging serves as a platform for health care providers to stimulate patient engagement and self-management during a very disruptive obstetric complication.

Upon diagnosis of gestational diabetes mellitus, patients are bewildered as to how to alter lifestyle habits, shop, and prepare for meals, let alone monitor blood glucose to keep their child safe. Unlike an office visit, secure messaging allows health care providers to instantly explain the diagnosis and send patient-education tools, guides, and even YouTube instructions so the patient can immediately launch into self-care management action. This patient action empowers patients to use secure messaging to maintain close communication with the health care providers. Secure messaging solidifies diabetes self-management for weekly review of blood glucose in order to determine the need for insulin therapy. Another challenge in pregnancy if insulin therapy is necessary is for patients to continue to engage in health restoration for themselves and their child. Patient education on insulin administration can be provided via telemedicine or office visits, with weekly glucose review for any insulin adjustment—all through secure messaging for the remainder of the pregnancy. Though the literature has demonstrated telemedicine and mobile health as modalities to improve diabetes control, evidence for the significance of clinical metrics for patient satisfaction with secure messaging is scant, although it has been shown to cause no harm. There is need for more research in this area.

It is clear that patient portals provide an additional avenue for patient-provider communication; however, the disparities found in this paper show that the portal is not equally accessible to several sociodemographic groups.

An early obstetrics encounter is the best time to introduce patients to the value and usefulness of the patient portal. Often, patients need assistance with setting up the patient portal. A mixed methods study analyzing factors affecting patient portal use among pregnant women with low income demonstrated that 33% of participants did not use the portal because they were unsure how to use it [26]. A practice message is always good to send while in the obstetrics office so the patient is comfortable with the EMR messaging application. Discussion of what will be available to the patient, such as lab results and appointment reminders, can lead to discussing the benefits of sharing blood glucose readings on a weekly basis. This can be crucial for medication and nutrition adjustment to improve diabetes self-care during pregnancy.

It is apparent that there is a need for innovative strategies and interventions aimed at enrolling Medicaid beneficiaries, those in poverty, young patients, and racial/ethnic minorities. With increased mobile phone use rates among women with low income, there is potential to increase portal use through interventions aimed at portal use via mobile apps.

### Limitations

Given that this is a retrospective study, the associations made in this study can not be assumed to be causal, and there is potential for unmeasured confounding. For example, this study did not have direct measures for health literacy, internet access, education level, or self-care behaviors, which may play a role in portal use. Additionally, although this sample was large and diverse, patients in this study received care at a large academic tertiary care center and findings may not be fully generalizable to other health settings. Furthermore, portal enrollment was not specifically studied with this data set, which is a parameter that has potential to highlight further sociodemographic and clinical disparities in portal use. As stated in the Methods section, our data did not permit identification of “threads” across multiple messages on the same issue, which would require natural language processing. This could be an important next step in messaging research.

### Conclusion

Portal use has major implications for redesigning obstetric delivery systems. This study documents that portal use and messaging frequency at our center have significantly increased in recent years, especially after the COVID-19 pandemic. Obstetric patients with high-risk chronic conditions are now more likely to use patient portals and to message frequently after the pandemic. However, sociodemographic disparities continue to exist fo portal use and intensity. Patient portals have the ability to encourage patient engagement and improve patient-provider communication and shared decision-making. Inclusive health literacy strategies developed for obstetric patients, such as training for and encouragement of portal use, may be a strategy to reduce disparities and improve outcomes. Future studies should focus on evaluating health and digital literacy interventions to address disparities in portal use.

## Abbreviations

EMR: electronic medical record
HITECH: Health Information Technology for Economic and Clinical Health
*ICD-10*: *International Classification of Diseases, Tenth Revision*
STROBE: Strengthening the Reporting of Observational Studies in Epidemiology
ZCTA: zip code tabulation area
